# 4 Risk Association Between Race and Complications Following Burn

**DOI:** 10.1093/jbcr/irac012.008

**Published:** 2022-03-23

**Authors:** Maria Haseem, Kendall Wermine, Sunny Gotewal, Tsola A Efejuku, Shivan N Chokshi, Lyndon G Huang, Jasmine M Chaij, Giovanna De La Tejera, Kassandra K Corona, George Golovko, Juquan Song, Steven E Wolf, Amina El Ayadi

**Affiliations:** University of Texas Medical Branch, Galveston, Texas; University of Texas Medical Branch, Galveston, Texas; University of Texas Medical Branch, Galveston, Texas; University of Texas Medical Branch, Galveston, Texas; University of Texas Medical Branch at Galveston, Galveston, Texas; University of Texas Medical Branch, Galveston, Texas; University of Texas Medical Branch, Galveston, Texas; University of Texas Medical Branch, Galveston, Texas; University of Texas Medical Branch, Galveston, Texas; University of Texas Medical Branch at Galveston, Galveston, Texas; University of Texas Medical Branch, Galveston, Texas; University of Texas Medical Branch at Galveston, Galveston, Texas; University of Texas Medical Branch at Galveston, Galveston, Texas

## Abstract

**Introduction:**

Racial minorities have been recognized to experience worse health outcomes for many medical conditions. However, it is not clear if these outcomes are associated with pre-existing conditions or the quality of care those patients receive. In burns, little information on the relationship between race and burn comorbidities is available. This study examines the risk association between race and burn complications, such as pneumonia, sepsis, and ARDS while controlling for common comorbidities that affect burn recovery such as diabetes and hypertension.

**Methods:**

Burn patient cohorts were identified by ICD10 codes for burn injury using TriNetX, a federated network of real-world data. The cohorts were stratified by race and balanced in terms of age at index, gender, BMI, and pre-existing comorbidities such as diabetes and hypertension. The following post burn outcomes were selected for analysis: renal failure, cardiovascular disease, sepsis, ARDS, graft complication, pneumonia, ICU admit, respiratory failure, hypertrophic scarring (HTS), hyperglycemia, and mortality. A measure of association analysis was performed to compare risk outcomes in black vs. white burn patients. Statistical significance was set at p< 0.05. The same cohorts were analyzed for treatment pathways to compare critical care billing CPT codes for the amount of time seen by a physician: Critical Care and Evaluation, first 30-74 minutes, and Critical Care and Evaluation, each additional 30 minutes.

**Results:**

The balanced patient cohorts comprised 78,974 patients per cohort. Black patients experience a positive relative risk ratio (RR) to renal failure (p < 0.0001, RR = 1.372, 95% CI: 1.314-1.435), cardiovascular disease (p < 0.0001, RR = 1.115, CI: 1.08-1.15), sepsis (p < 0.0071 RR = 1.081, CI: 1.021-1.144), and ARDS (p < 0.0010, RR = 1.287 CI: 1.107-1.496) following burn injury. However, black patients experience a negative risk ratio to mortality (p < 0.0006, RR = 0.935, CI: 0.89-0.982) and pneumonia (p < 0.0014, RR = 0.937, CI: 0.901-0.975). The risk ratio was not significant for outcomes between black and white burn patients for respiratory failure, HTS, hyperglycemia, and ICU admit. Analysis of treatment pathways did not show significant differences in Critical Care and Evaluation billing between the two races.

**Conclusions:**

Black burn patients are more likely to experience renal failure, cardiovascular disease, sepsis, and ARDS compared to white burn patients despite controlling for common comorbidities. They are less likely to experience pneumonia and mortality.

